# Adaptability, resilience and environmental buffering in European Refugia during the Late Pleistocene: Insights from La Riera Cave (Asturias, Cantabria, Spain)

**DOI:** 10.1038/s41598-020-57715-2

**Published:** 2020-01-27

**Authors:** Jennifer R. Jones, Ana B. Marín-Arroyo, Lawrence G. Straus, Michael P. Richards

**Affiliations:** 1 Instituto Internacional de Investigaciones Prehistóricas de Cantabria, (Universidad de Cantabria, Santander, Gobierno de Cantabria), Santander, 39005 Spain; 2 Department of Anthropology, MSC01 1040, University of New Mexico, Albuquerque, New Mexico United States of America; 3 Department of Archaeology, Simon Fraser University, Burnaby, British Columbia Canada

**Keywords:** Palaeoclimate, Evolutionary ecology, Stable isotope analysis, Climate-change adaptation

## Abstract

The Upper Palaeolithic in Europe was a time of extensive climatic changes that impacted on the survival and distribution of human populations. During the Late Glacial Maximum (LGM), southern European peninsulas were refugia for flora, fauna, and human groups. One of these refugia, the Cantabrian region (northern Atlantic Spain), was intensively occupied throughout the Upper Palaeolithic. Characterising how climatic events were expressed in local environments is crucial to understand human and animal survival. La Riera Cave (Asturias) has a rich geo-cultural sequence dating between 20.5kyr BP to 6.5kyr BP and represents an ideal location in which to explore this. Stable isotope analysis of red deer and ibex is used alongside other environmental and climatic proxies to reconstruct Late Upper Palaeolithic conditions. Results show that during the LGM, ibex adapted their niche to survive, and became a major prey species for humans. The diverse environmental opportunities offered in the high-relief and coastal environs of La Riera may help to explain the high human population levels in the Cantabrian Region throughout the Late Upper Palaeolithic. Despite fluctuating conditions, herbivores and humans had the flexibility and resilience to adapt, demonstrating the importance of southern European refugia for the survival of different species.

## Introduction

**Figure 1:**
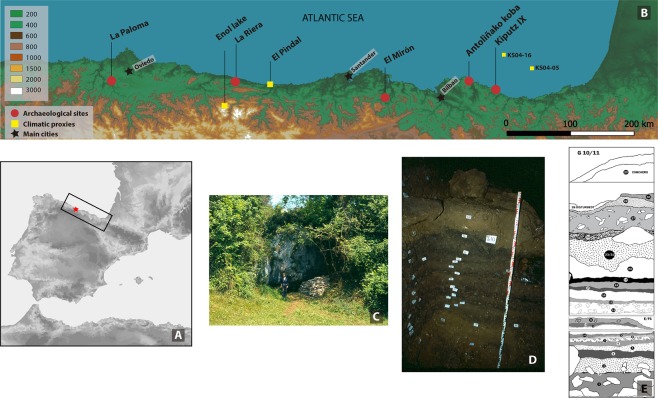
Location of La Riera cCave within the Cantabrian Region, Northern Spain (Panel A) with other contemporary archaeological sites, together with available environmental and climatic proxy records, referred in the text (Panel B). Panel C shows the outside view of La Riera Cave in the summer of 1976 and Panel D displays the stratigraphy sequence of squares G/H at the site (Photos by L.G. Straus). Panel E is a re-drawing of the stratigraphy at La Riera (based on the original version by G.A. Clarke^82^).

The settlement of Europe during the Last Glacial (MIS2) period (c.30-10kyr BP) was directly affected by the climate which resulted in cycles of expansion and contraction of populations, linked to the ebb and flow of ice sheets^1–3^. Central and northern Europe during the Last Glacial Maximum (LGM) (c. 21-18kyr BP) were mostly covered by ice sheets or polar desert that pushed populations into the south, with Iberia and southwestern France, Italy and the Balkan peninsulas acting as refugial regions. Within these locations less drastic temperatures, associated with milder conditions, facilitated survival of a variety of plant and animal species^4–13^. Of these areas, the Cantabrian region, which occupies the northern Atlantic coast of the Iberian Peninsula (Fig. 1) has been demonstrated to have been a genetic refuge for salmon^14^, red deer^15^ and humans^16,17^. At least 55 archaeological sites dating to the Solutrean (24kyr BP) have been recorded in the Vasco-Cantabrian region, concurrent with a large increase in the number archaeological occupation levels recorded within many sites^13,18^, indicative of a shift of surviving populations in Europe to the region^10,11,19–21^. During the Magdalenian (c. 17-11.5kyr BP), a further population expansion is observed with a dramatic increase in the number of archaeological sites recorded, linked to ameliorated climatic conditions^21,22^ as identified in proxies such as ice and marine cores. This region has been described as a “dynamic cultural center in the Upper Palaeolithic world of western Europe”^23^, as reflected by and, in part, due to its extensive and rich cave art and portable art. These phenomena clearly demonstrate the importance of the region. Characterisation of how global climatic events that occurred throughout this period were expressed locally, and the impact that they had on ecological niches, adaptability and resilience of populations, are key to understanding why this region functioned as a refugium throughout the Late Pleistocene, enabling the survival of humanity throughout these climatically challenging conditions.

Ice core evidence from Greenland^24,25^ provides information on larger climatic events that occurred throughout MIS2/1, including the Last Glacial Maximum, Heinrich 1 event, Greenland Interstadial 1, Younger Dryas, and eventually the onset of the Holocene. The integration of ice and marine cores with terrestrial records has helped to refine the stratigraphy of these events^25–27^. These larger scale climatic records are physically far removed from conditions experienced on regional and local levels in northern Spain. Speleothem records^28^, lake sediments^29,30^, ostracod evidence from the continental shelf^31^ reflect data closer to study areas, enabling regional-scale environmental reconstructions, but these are still distant from the sites occupied by human groups. More localised environmental indicators such as pollen, found within cave sites, can inform on past vegetation^32^ and have been analysed in Asturian cave sites^33,34^ but must be used with caution as they can be subject to taphonomic and diagenetic alterations^35,36^. Both macro- and micro- mammal remains are ubiquitously found on these sites^37–43^ and are often less affected by taphonomic and sampling biases than other environmental proxies.

Bone collagen δ^13^C and δ^15^N analyses have been abundantly applied in European settings to explore palaeoclimates and palaeoenvironments^44–47^ and has yielded successful results for the Middle and Upper Palaeolithic of northern Spain^26,48–52^. Analysis of δ^13^C and δ^15^N in bone collagen is a powerful tool for reconstructing palaeoenvironments, especially when combined with other available environmental proxies^53^. Carbon and nitrogen in animal bones derives directly from consumed diet^54^, with a fractionation effect^55^, and shows long-term dietary behaviour of animals during the period of bone growth, typically representing the lifetime of each individual animal^56,57^.

A multitude of climatic and environmental factors can affect the δ^13^C and δ^15^N values of plants, and thus the animals that consume them. Regarding δ^13^C values, temperature has been seen to affect values both positively and negatively^58^, although the δ^13^C values of plants appear to have a greater correlation with mean annual precipitation than mean annual temperature, and typically higher levels of precipitation result in lower plant δ^13^C values^59,60^. Increased tree cover can result in lower δ^13^C values in relation to more open environments linked to a combination of closed carbon cycling of CO_2_ under the forest’s canopy limiting atmospheric exchange and recycling of CO_2_ from decomposing leaf matter^61–64^. Plants growing at higher altitude can typically produce elevated δ^13^C values linked to decreased temperatures and changing CO_2_ partial pressure^65^, and has been observed archaeologically to produce elevated δ^13^C in the fauna that reside at higher altitudes^66^. Global scale conditions such as a change in atmospheric CO_2_ levels can also impact on δ^13^C values of plants^67,68^, and thus be expressed in fauna. Larger-scale drivers and climatic events such as this would be expected to be observable in multiple species within a site, and typically should be observed across different geographical locations.

A negative correlation between plant δ^15^N values and annual precipitation has been observed in a variety of ecosystems^69–71^. Higher temperatures and lower water availability are thought to produce higher animal δ^15^N values in hot/arid potentially due to physiological stress due to heat and lack of water^72–74^, although research has suggested that this changes could be linked to dietary shifts associated with these conditions^75,76^. Colder temperatures are frequently associated with lower δ^15^N values^69^ due to reduced activity of nitrogen fixing mycorrhizae^77,78^. There is a negative relationship between soil δ^15^N values and altitude^79^. Different geographical locations, can have exhibit variation in baseline δ^15^N due to the nature of soil types within a given region, which affects plant communities, and nitrogen-fixing mycorrhizae present^80^.

In addition to environmental and atmospheric drivers affecting plants (and their consumers), changing environmental conditions can also affect faunal behaviour, in terms of the dietary and habitat choices of animals. Animal diets may shift to include different plant species consumed, or parts of plants, with differing isotopic signatures resulting from plant physiological variations^58^. Due to the complex processes influencing how localised and regional conditions are expressed within the isotopic values of plants, and the animals that consume them, temporal trends in δ^13^C and δ^15^N values can be expressed differently between regions, and changing conditions do not always affect every species in the same way^46,81^. Wider environmental indicators can be used to facilitate interpretations of isotopic results to understand the trends that are visible.

When applied to bones with evidence of anthropogenic modifications (e.g., exhibiting cut marks, marrow cracking, hammer blows), bone collagen δ^13^C and δ^15^N analysis can provide direct evidence of past environments during the times when these animals were consumed and is one of the few proxies that can inform on how climatic events were experienced by humans and animals on a local scale. This study combines newly generated δ^13^C and δ^15^N evidence from La Riera, one of the most important, and well-published Upper Palaeolithic sites in the Cantabrian region with wider climatic and environmental proxies from local, regional and global records to explore environmental changes throughout the archaeological sequence to understand how humans and animals adapted and became resilient to the changing conditions that they faced.

## La Riera Cave

La Riera (Posada de Llanes, Asturias) (Fig. 1) was discovered and first excavated by Conde de la Vega del Sella in 1916 and was re-excavated by L.G. Straus and G.A. Clark in 1976–79. All materials discussed in this paper relate to the findings from the 70 s excavations. The site contains a long sequence of archaeologically rich levels dating from the Solutrean to the Asturian (Mesolithic) cultural periods, C14-dated between 20–6.5kyr BP^82^ (Table 1). La Rieralies c. 30 m above sea level on the south-facing edge of a low ridge on the narrow coastal plain facing the steep, nearby cliffs of the 800 m a.s.l. Sierra de Cuera, a coastal mountain range north of the massive Picos de Europa chain. Currently located 2 km from the shore, during the LGM, when sea level was approximately 120 m lower, the site was around 10 km from the shore, roughly two hours walk from the Late Glacial littoral^82^. The location of the site means that hunting areas available included the montane habitats of the Sierra, the low, rolling coastal plain, the valleys of the small Calabres and Bedón rivers, and their estuaries offering a selection of wild terrestrial, marine, and riverine resources^82^

**Table 1:** Radiocarbon (and AAR) dates of the archaeological levels sampled in this study, including the number of specimens per species analysed for bone collagen δ^13^C and δ^15^N values. Where direct dates for a level were not available, dating evidence from adjacent levels has been included. Shell C14 dates adjusted upward by 105 years for the Holocene marine reservoir effect, per Soares *et al*.^143^.

Level	Cultural attribution	Lab number	Material	Dating method	^14^C date	±	Red deer n	Ibex n	Total n
*29*	Asturian	GaK-3046^82^	Charcoal	C14	6500	200	7	4	11
		RIE-1^88^	Shell	AAR	7516	588			
		GaK-2909^82^	Charcoal	C14	8650	300			
*28*	Azilian	Q-2933^87^	Shell	C14	9125	90	7	3	10
*27*		BM-1494^82^	Bone	C14	10630	120	7	7	14
		Q-2935^87^	Shell	C14	11285	70			
		UCR-1275D^82^	Bone	C14	12270	400			
24	Upper Magdalenian	GaK-6982^82^	Charcoal	C14	10890	430	7	3	10
		Q-2926^87^	Shell	C14	11775	75			
		UGAMS-27525^89^	Bone	AMS	13530	35			
*23*		UGAMS-27526^89^	Bone	AMS	15120	40	7	4	11
		Q-2932^87^	Shell	C14	13095	110			
		UCR-1274D^87^	Bone	C14	12620	300			
*19*	Lower Magdalenian	Q-2116^82^	Charcoal	C14	15230	300	7	7	14
		Q-2110^82^	Charcoal	C14	15520	350			
		GaK-6448^82^	Charcoal	C14	16420	430			
*18*		Q2936^87^	Shell	C14	15885	150	7	7	14
*15*	Solutrean	UCR-1272A^82^	Bone	C14	17225	350	—	—	—
14		Q2927^87^	Shell	C14	16705	105	—	—	—
13		—	—	—	—	—	8	7	15
*12*		GaK-6446^82^	Charcoal	C14	17210	350	—	—	—
*11*		—	—	—	—	—	7	7	14
*10*		GaK-6447^82^	Charcoal	C14	19820	390	—	—	—
*7*		Q2934^87^	Shell	C14	20295	210	7	7	15
						Totals	71	56	128

.

To date, La Riera remains one of the most extensively studied sites in the Cantabrian Region, and was a landmark study, as it provided detailed scientific analysis, including extensive analyses of palaeoenvironmental remains including: pollen records, deposited sediments, bird bones, macro- and micro-fauna, and even a pioneering analysis of mollusc shell isotopes^34,37,83–86^. Several series of radiocarbon dates^82,87,88^ were undertaken both by the excavators and subsequent researchers, and two new dates for Levels 23 and 24^89^ run for this study have resolved some previous dating discrepancies in chronologies at the site (Table 1).

Although not permanently occupied, La Riera was repeatedly visited and inhabited, during different seasons, by human groups throughout the Upper Palaeolithic and Mesolithic and was an important hunting and gathering base^82^. La Riera was as part of a network of Upper Palaeolithic sites across the Cantabrian region. The Solutrean (Levels 4 to 17) dates to between 20.5-17kyr BP, and the earliest Solutrean occupations are interpreted as representing mainly a hunting camp for the procurement of red deer and mainly ibex^90^. The site then became a multi-purpose residential camp, still exploiting large numbers of red deer and ibex with fish and shellfish also being collected^90^. The Lower Magdalenian (Levels 18–20) date to between 17-14kyr BP and the Middle and Upper Magdalenian (Levels 21–26) span between 14.5-13kyr BP. The Azilian, a form of local Mesolithic industry, (Levels 27–28) falls between around 10.5-9kyr BP, and at the end of the sequence, Level 29 (attributed to the Asturian, also a local Mesolithic industry) is a shell midden (‘conchero’) formed between roughly 9-6.5kyr BP. Towards the end of the sequence, faunal evidence suggests stricter bi-seasonality, with occupation focussed on the summer and winter^90^. The faunal record for La Riera shows evidence of intensive carcass processing, including marrow extraction from the low-yield phalanges and mandibles of red deer, and large numbers of individuals per level were recorded^37^ despite the relative small portion of the site excavated, reflecting intensive hunting strategies. Most notably from the Upper Magdalenian levels onwards (Level 24–29) there is increasing evidence of slaughtering *Cervus* hinds and their young, alongside a more diverse faunal suite, including greater frequencies of birds, fish, and marine molluscs^37^, suggesting increasing levels of dietary diversification and further intensification of diet throughout the Upper Palaeolithic.

La Riera is 26 km west of coastal El Pindal cave, famous for its Magdalenian cave art and yielding a speleothem record now used as a palaeoenvironmental proxy^28,91,92^ (Fig. 1). Lake Enol, situated at c. 1000 m above present sea level about 20 km WSW in the Picos de Europa of Asturias (Fig. 1) contains valuable lake sediment information regarding temperatures throughout the Late Pleistocene^27,30^. The relative proximity of La Riera to sites with additional environmental proxies, facilitates a more nuanced interpretation of the past conditions experienced by the human groups that occupied La Riera.

## Results and Discussion

### Overview of the δ^13^C and δ^15^N data

In total 128 specimens (72 red deer and 56 ibex) were analysed for δ^13^C and δ^15^N in bone collagen from 10 different archaeological levels at La Riera, from the Solutrean to the Asturian period, covering a temporal span of almost 15kyr BP (Table 1). Summary statistics of these results are shown in Table 2. Preservation at the site was exceptional and collagen was successfully extracted and analysed from 121 specimens (94.5%). All specimens analysed had atomic C:N ratios between 3.2–3.5, falling within the accepted ranges for well-preserved collagen^93^, with 110 specimens of these having ratios between 3.2–3.4, adhering to more rigid criteria of Van Klinken^94^. The vast majority of specimens had >35%C and %N values between 10–16%, and 109 of the specimens collagen yields were >1% (based only on the >30Ka fraction, rather than total collagen), indicative of well-preserved *in vivo* collagen^94^, as detailed in the Supplementary Information (SI1).

**Table 2:** Summary statistics of red deer and ibex δ^13^C and δ^15^N values from levels sampled at La Riera.

Level	δ^13^C						δ^15^N				
	n	Mean	Min.	Max.	Range	1σ	Mean	Min.	Max.	Range	1σ
	*Red deer (Cervus elaphus)*										
7	7	−20.2	−20.8	−19.6	1.2	0.5	4.3	3.4	5.8	2.4	1.0
11	7	−20.9	−21.2	−20.4	0.8	0.3	3.7	3.1	4.3	1.2	0.4
13	7	−20.6	−21.2	−20.2	1.0	0.4	3.5	3.0	4.3	1.3	0.5
18	7	−20.7	−21.1	−20.5	0.6	0.2	4.0	3.6	4.4	0.8	0.3
19	7	−20.8	−21.3	−20.7	0.6	0.2	4.2	3.7	4.8	1.1	0.4
23	7	−20.8	−21.0	−20.0	1.0	0.4	3.9	2.8	5.3	2.5	0.7
24	7	−20.2	−20.8	−19.6	1.2	0.5	4.3	3.4	5.8	2.4	1.0
27	6	−21.2	−21.5	−20.9	0.6	0.2	4.5	3.8	5.1	1.3	0.5
28	5	−21.0	−21.3	−20.7	0.6	0.2	3.8	3.5	4.3	0.8	0.3
29	7	−21.1	−21.6	−20.1	1.5	0.5	4.1	2.4	5.1	2.7	1.0
	*Spanish ibex (Capra pyrenaica)*										
7	5	−20.5	−21.3	−19.9	1.4	0.6	3.0	2.3	3.5	1.2	0.5
11	5	−20.1	−20.5	−19.5	1.0	0.4	2.5	2.0	3.0	1.0	0.4
13	6	−20.2	−20.4	−20.0	0.4	0.2	2.7	2.1	3.8	1.7	0.6
18	7	−20.6	−21.4	−20.0	1.4	0.5	4.0	2.6	5.3	2.7	0.8
19	6	−20.6	−21.7	−19.9	1.8	0.6	4.0	3.6	4.4	0.8	0.3
23	4	−20.6	−21.4	−20.3	1.1	0.5	3.6	2.9	4.5	1.6	0.7
24	3	−19.9	−19.9	−19.8	0.1	0.1	4.6	4.0	5.2	1.2	0.6
27	7	−21.4	−21.9	−20.2	1.7	0.6	4.4	3.1	4.9	1.8	0.7
28	3	−20.7	−21.1	−20.4	0.7	0.4	3.5	3.2	3.9	0.7	0.4
29	4	−20.9	−21.7	−20.3	1.4	0.6	3.8	2.7	5.2	2.5	1.0

A series of trends were observable throughout the sequence due to changing δ^13^C and δ^15^N values of the species analysed. To facilitate discussion the sequence has been divided into interpretative zones (Z1-Z5) based on key characteristics of the data including; changing statistical relationships of the δ^13^C or δ^15^N values of each species, increases/or decreases in the isotopic values of both species, observable by statistically significant differences between archaeological levels, and in one instance notably large ranges in isotopic values of both species observed. A thorough explanation of the key characteristics of each interpretative zone is as follows: Zone (Z1), at the bottom of the stratigraphic sequence, dating to the Solutrean, is characterised by partitioning of the red deer and the ibex, with the latter having 15-N depleted values. Zone 2 (Z2, Middle into Upper Magdalenian) is marked by an increase in the δ^15^N value of the ibex, followed by stability, in both δ^13^C and δ^15^N values. Zone 3 (Z3, Upper Magdalenian) is differentiated by a small increase in the δ^13^C values of both species observed between Levels 23 and 24. Zone 4 (Z4, Azilian) is marked by a dramatic decrease in the δ^13^C values in both species. Finally, Zone 5 (Z5, Asturian) exhibits similar δ^13^C and δ^15^N values to the preceding levels (27 and 28), but is marked as being different due to a larger range in the δ^15^N values of both the red deer and ibex in comparison to the rest of the sequence. The isotope data are correlated with environmental proxies on a local, regional and global scale to determine how trends observed relate to wider environmental and climatic trends. Raw data are provided in SI1–10.

The results and interpretation are framed by exploring the patterns in the stable isotope record at La Riera, outlining the conditions experienced continentally within the region and the implications that this had for human groups, before being drawn together in the final discussion.

### Zone 1: The Solutrean (Levels 7-13: 20- 17kyr BP)

**Figure 2:**
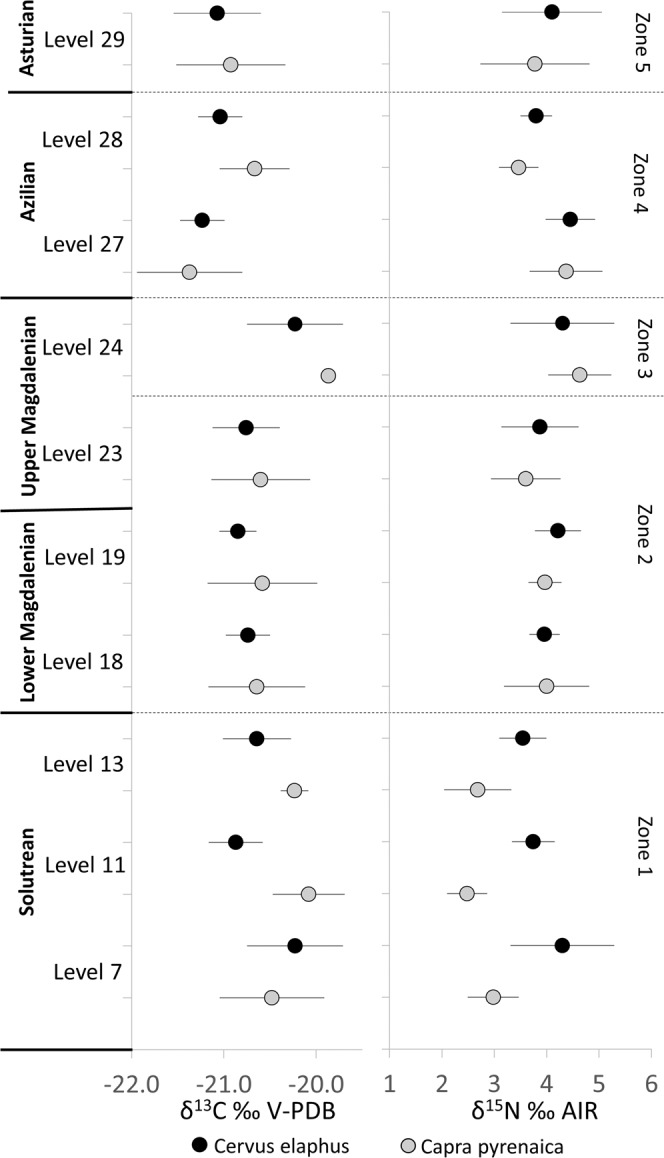
Mean δ^13^C values (left) and δ^15^N values (right) of red deer (denoted by black circles) and ibex (denoted by grey circles) from the Upper Palaeolithic levels sampled throughout the sequence at La Riera. Error bars show 1σ.

The Solutrean levels (7, 11 and 13) exhibit a marked difference in the δ^15^N values of ibex and red deer, with red deer having consistently higher δ^15^N values by at least 1‰, and no overlap in standard deviation is seen between the two species (Fig. 2). A statistically significant difference in in the δ^15^N was seen between the two populations for each of these levels (Table 3). From Level 18 onwards, no difference in the δ^15^N values of the two species was observed (Fig. 2, Table 3). The ibex have the lowest range in δ^15^N values seen within the sequence at La Riera during this time (Table 2).

**Table 3:** Comparisons of red deer and Spanish ibex δ^13^C values and δ^15^N values, within each archaeological level sampled, to determine similarities and differences between the dietary niches of both species. Numbers in bold denote statistically significant results.

Level	7	11	13	18	19	23	24	27	28	29
δ^13^C	0.36	**0.01**	0.07	0.81	0.06	0.23	1	0.17	0.17	0.59
δ^15^N	**0.02**	**0.00**	**0.04**	0.93	0.57	0.45	0.52	0.94	0.37	0.92

The δ^13^C values show little difference between the two species. Only in Level 11 were the mean ibex values 0.7‰ lower than the red deer (Table 1; Fig. 2), and the populations were statistically different (*p* = 0.01) (Table 3). The mean δ^13^C value of ibex was also slightly higher within Level 13 (Table 2), although there was no statistically significant difference (*p* = 0.07) (Table 3). There is a decrease in δ^13^C values of the deer between levels 7 and 11, which was statistically significant (*p* = 0.02) (Table 4).

**Table 4:** Independent comparisons of red deer (upper rows) and ibex (lower rows) δ^13^C values and δ^15^N values between consecutive archaeological levels sampled to explore temporal changes. Numbers in bold denote statistically significant results.

Species	Levels Compared	7&11	11&13	13&18	18&19	19&23	23&24	24&27	27&28	28&29
Red deer	δ^13^C	**0.02**	0.25	0.61	0.34	0.70	**0.03**	**0.00**	0.23	0.62
	δ^15^N	0.48	0.37	0.07	0.37	0.20	0.61	0.43	**0.03**	0.33
Ibex	δ^13^C	0.40	0.58	0.37	0.93	0.51	**0.04**	**0.03**	0.15	0.86
	δ^15^N	0.17	0.71	**0.02**	0.58	0.24	0.11	0.69	0.15	0.86

**Figure 3:**
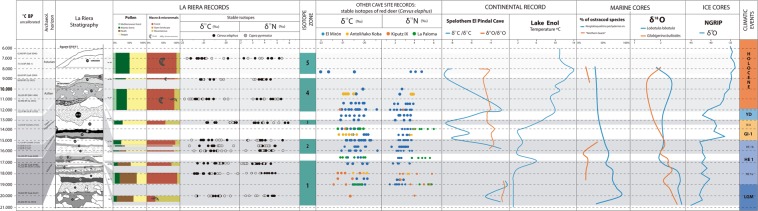
Multi-scale environmental and climatic comparisons throughout the Upper Palaeolithic on a local, regional and global level. Stable isotope δ^13^C and δ^15^N values of red deer and ibex sampled at La Riera are shown alongside additional environmental proxies from the site^34,37^, and isotopic values from red deer across the Cantabrian Region^26,48,49,52^.Wider continental environmental indicators^28,92^, offshore proxies^31^ and NGRIP ice core evidence^25^, and climatic events^141^ are also displayed to further contextualise trends observed through time.

**Figure 4:**
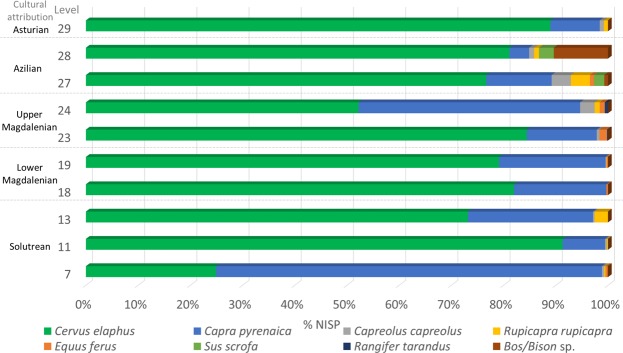
The %NISP of each of key prey species exploited within the sampled levels in this study showing the variation of each species through time, based on data produced by Altuna^37^.

The difference in δ^15^N values indicates niche partitioning of ibex and red deer. Pollen records available from Levels 6–15 indicate a predominance of *Ericacea* (heather/heath plants), accounting for between 60–90% of the pollen recorded, and little tree cover, with arboreal pollen accounting for <5%^34^ (Fig. 3). *Microtus oeconomus*, a species adapted to cold environments, is identified in Level 7^37^. Montane species (ibex) are exploited in greater quantities (74% of NISP in Level 7, declining to 30% and 15% throughout this zone), although red deer are still present in great numbers (25% in Level 7, increasing to 80–90% in Levels 11 and 13 this zone)^37^ (Figs. 3 and 4).

Within the Solutrean Z1 lower δ^15^N values in the red deer were noted at El Mirón in comparison to values within Z2 from the site (*p* = 0.00) (Table 5; Fig. 3), which could suggest colder, drier conditions during this time. Typically El Mirón δ^15^N values are lower than those seen at La Riera (mean values range between 2.4–2.8‰ in Z1-4), indicative of wider, baseline differences between these two geographically distinct locations (Table 5: Fig. 3). Antoliñako Koba δ^13^C values were higher in Z1, compared to those seen later on in the sequence (Table 5; Fig. 3) which could indicate colder/drier conditions.

**Table 5:** Summary statistics to compare δ^13^C and δ^15^N values of red deer for each isotopic zone at La Riera with contemporary results from the wider the wider Cantabrian Region sites of El Mirón (Cantabria)^52^, Kiputz IX (Gipuzkoa)^48^, Antoliñako Koba (Bizkaia)^26^ and La Paloma (Asturias)^49^, where data are available (see Supplementary SI6).

Archaeological site	δ^13^C values							δ^15^N values				
	Isotope Zone	N	Mean	Min	Max	Range	1σ	Mean	Min	Max	Range	1σ
La Riera	1	20	−20.7	−21.2	−20.2	1.0	0.3	3.7	3.0	4.3	1.3	0.4
El Mirón		16	−20.3	−20.7	−19.9	0.8	0.3	2.4	1.6	3.4	1.8	0.4
La Paloma		6	−20.3	−20.5	−20.2	0.4	0.2	5.2	4.4	5.5	1.1	0.4
Antoliñako Koba		3	−20.2	−20.6	−20.0	0.6	0.3	3.3	3.3	3.4	0.1	0.1
Kiputz IX		11	−20.9	−21.5	−20.3	1.2	0.3	3.9	2.5	5.7	3.2	1.2
La Riera	2	21	−20.8	−21.3	−20.0	1.2	0.3	4.0	2.8	5.3	2.5	0.5
El Mirón		40	−20.4	−21.2	−19.6	1.6	0.3	2.8	1.7	3.7	2.0	0.4
Kiputz IX		1	−21.3	—	—	—	—	3.5	—	—	—	—
Kiputz IX		1	−20.9	—	—	—	—	5.5	—	—	—	—
La Riera	3	7	−20.2	−20.8	−19.7	1.2	0.5	4.3	3.4	5.8	2.4	1.0
El Mirón		3	−20.6	−21.0	−20.4	0.6	0.3	2.7	2.1	3.2	1.1	0.6
La Riera	4	14	−21.1	−21.5	−20.8	0.7	0.2	4.1	3.4	5.1	1.7	0.5
El Mirón		26	−20.4	−21.0	−19.6	1.4	0.3	2.7	0.6	3.5	2.9	0.7
Antoliñako Koba		5	−20.9	−21.0	−20.7	0.3	0.1	3.4	3.3	3.5	0.1	0.1
Kiputz IX		1	−21.1	—	—	—	—	2.8	—	—	—	—
La Riera	5	7	−21.1	−21.6	−20.1	1.5	0.5	4.1	2.4	5.1	2.7	1.0
El Mirón		3	−21.4	−22.0	−20.5	1.5	0.8	4.0	3.5	4.5	1.0	0.5

The water temperature estimates for Lake Enol are calculated at 1 °C between 20-17kyr BP, (Fig. 3), with no change registered in the Ca precipitation record, although during cooler times, this record becomes less precise^27^. Speleothem evidence from El Pindal cave shows some of the lowest δ^13^C values and a hiatus in growth between 18.2-15.4kyr BP^28^ (Fig. 3). This interruption is interpreted as belonging to one of the coldest and potentially relatively driest parts of the sequence^28^ (Fig. 3). Lower δ^18^O values in the NGRIP record at 20kyr BP indicate that this part of the sequence corresponds to the end of the LGM, which concluded at c. 19kyr BP, when the onset of Heinrich 1 event marks a climatic improvement^24^, with a slight increase in δ^18^O values (Fig. 3). The ostracod evidence, from the Basque continental shelf, also attests to generally cooler sea temperatures^31^ (Fig. 3).

#### Interpretation

The Solutrean was a cold period, representing the latter stages of the LGM and start of the Heinrich 1 event, as seen continentally and in ice core and offshore marine records. The consistently lower δ^15^N values of ibex during this time, in comparison to the red deer (and in relation to the ibex analysed from later in the sequence) is distinctive at La Riera. Higher altitudes are associated with depleted δ^15^N values of soils and plants^79^, and it has been proposed that animals feeding at altitudes may have produced lower δ^15^N values of faunal, as seen in Northern Europe, and at the Cataluña site of Balma Guilanya during the 20-10kyr BP^95^. Typically higher altitudes are also associated with elevated δ^13^C values in plants^65^, which has been archaeologically observable within ibex at the site of Valdegoba in central Northern Spain^66^, and is not evident at La Riera. There is no difference between the ibex and the red deer δ^13^C values throughout the sequence, suggesting that both species are feeding at isotopically similar altitudes, making this less likely to be the cause for the lower values of the ibex. Furthermore, studies of Mediterranean vegetation indicate that both species density and diversity declines with altitude^96^, suggesting that scaling higher altitudes would be less profitable for ibex, especially during the hostile conditions of the Solutrean. Thus, alternative drivers are responsible for the difference in δ^15^N values of the two species during the Solutrean.

The lower δ^15^N may be linked to changing patterns of vegetation consumption, resulting in the niche of the ibex being altered to adapt to the cold conditions of the Solutrean. The lower ibex δ^15^N values occur alongside increased quantities of *Ericaceaous* (heath) plants^34^ (Fig. 3). *Ericaceaous* plants typically occupy regions with lower quality, acidic soils and have mycorrhiza which enhance their ability to absorb nitrogen from soils^97^. The larger proportions of ericaceous plants suggest more hostile environmental conditions were experienced during these levels. Studies of recent red deer and ovicaprids in Scotland have shown that red deer can digest heather better than ovicaprids^98^. Ecological research into the feeding behaviour of ovicaprids and red deer in mosaic heath landscapes showed that the dietary selection of ovicaprids was heavily affected by the presence of heather, resulting in the two species consuming different diets^99^, and could explain the observed niche partitioning in these archaeological specimens during the Solutrean at the site. Also, ecological studies of ibex have demonstrated that they are pushed into alternative niches when competing with livestock^100^, and a similar effect might be observed when faced with red deer as a competitor. Red deer are an ecologically flexible species, thriving in open grasslands, in addition to forested environments^101–103^. Their combination of grazing and browsing behaviour means that they have great flexibility in their dietary behaviour, already observed archaeologically^104^, and thus may have been able to continue existing the ericaceous landscape that proved challenging to the ibex. Ibex are known to have a high plasticity in the feeding strategies depending on ecological constraints^105^, and it is possible that the ibex populations were being pushed away from the ericaceous vegetation communities of the ^15^N depleted heathlands and were consuming a different diet. The mobility of ibex reduces when food sources are scarcer and encounter rates are low^106^ and they have a reduced dietary breadth in the winter months^107^. Their high representation within the zooarchaeological record suggests their niche habitat was well-known to Solutrean hunters as a reliable and consistent source of animal protein and may explain the draw of people to La Riera during these colder stages.

### Zone 2: The Lower Magdalenian and Upper Magdalenian (Levels 18-23: 16-15kyr BP)

Mean δ^15^N values of ibex in Lower Magdalenian Level 18 are 1.3‰, higher than in Level 13 (Fig. 2, Table 2), and the populations were statistically different (*p* = 0.02; Table 3). This difference disappears after the formation of Level 13 and the values of red deer and ibex track each other relatively closely in mean values, ranges and standard deviations (Table 2), and the diet of both species becomes isotopically indistinguishable. An increase in mean δ^13^C values of red deer is seen between Levels 13 and 18, although this was not statistically significant (*p* = 0.07) (Table 4). Throughout the Lower Magdalenian and into the Upper Magdalenian (Levels 18, 19, 23) (16-15kyr BP), the δ^15^N and δ^13^C values of both the red deer and the ibex remain stable (Table 2, Fig. 2). Red deer and ibex populations within Levels 18–19 and Levels 19–23 were statistically similar (Table 4).

With the conclusion of the Solutrean, from Level 16 onwards, there was a large decrease in *Ericaceae* and heath was replaced by steppic vegetation (including *Liguliflorae, Tubuliflrae, Chenopodiaceae*, and *Artemisia*)^34^ (Fig. 3). Arboreal pollen increased slightly from before, but remains low, accounting for less than 10% of the total pollen record between Levels 16–23^34^. Lowland species (notably red deer) are the most commonly exploited game (70%), but with montane (steep, rocky habitat-preferring) species (predominantly ibex) also representing a valuable resource within these levels (Fig. 4)^37,38^.

The wider isotopic data from the Cantabrian Region red deer also show little temporal change in δ^13^C values in the Lower Magdalenian to the Upper Magdalenian (17-12kyr BP) (Table 5; Fig. 3). Within Z2, the El Mirón δ^13^C values indistinguishable from those seen in Z1 (*p* = 0.798) (Table 5). The mean δ^15^N value at El Mirón is 2.8‰ which is 0.4‰ higher than observed in Z1 (Table 5). Lake Enol temperatures of 2 °C are estimated during this period, warmer than previously observed^27^. This period in the NGRIP ice core record belongs to Heinrich 1b event, followed by Greenland Interstadial 1, which represents a warmer climate (Fig. 3). A decrease in the “Northern Guest” benthic foraminifera between 16-15kyr BP, indicate warmer sea bottom temperatures off the Basque shelf, and warmer seawater surface temperatures are suggested by the lower δ^18^O values and decline in *N. pachyderma sin*. %^32^.

#### Interpretation

Climatic amelioration is attested within the La Riera sequence by a shift in vegetation, that occurs with the change to GI1 signalled by a decline in heath plants and an increase in steppic plants, then suggesting a warmer and slightly drier environment. The δ^15^N values of the ibex increase in this zone, and they become isotopically indistinguishable from the red deer. Warmer conditions which may explain the increased δ^15^N values of the ibex, as a product of increased soil microbial activity^77,78^, although this would be expected to affect the red deer values also. The lack of covariance in the δ^15^N values of the La Riera red deer suggests that drivers behind this change are specific to the niche of the ibex. The shift in the ibex δ^15^N values may be linked to dietary change of the ibex related to the reduction in *Ericacea*, allowing the ibex could expand into regions that were previously less desirable, adapting their niches to suit these new environmental conditions. The consistency in the stable isotope values of both species throughout Levels 18–23 indicates that the environment was relatively stable within these levels, as is mirrored in continental stable isotope results across the region. The increase in red deer bones within the zooarchaeological assemblage suggests that there is a shift in hunting focus, associated within these milder conditions.

### Zone 3: Upper Magdalenian (Level 24:  13.5kyr BP)

During the Upper Magdalenian occupation of the site, between Levels 23 and 24, an increase in δ^13^C is observed, and mean values in both red deer and ibex are higher within Level 24 (Table 2, Fig. 2), which was statistically significant for both species (Table 4). A slight increase in δ^15^N values is visible for both species (Table 2, Fig. 2), although this was not statistically significant for red deer (*p* = 0.61) or ibex (*p* = 0.11) (Table 4). A stratigraphic hiatus was recorded between these two levels and new dating of ungulate bones from these levels (Table 1), revealed a 2,000-year gap between them, with Level 24 marking a restart in the archaeological sequence^82,89^.

The lack of thermophilic species in Level 24 in the palynological record suggests a cooler environment. There is a small increase in arboreal plants, steppic taxa continue to be represented, and generally tree cover remains low (Fig. 3) with ferns present only in the upper parts of level 24^34^. Previous interpretations of the level suggested that it was formed during a cold spell within the Allerød^34,82^. Reindeer was present within this level (Fig. 4)^37^ and sedimentary evidence indicated a colder environment than in the previous level^85^. There is evidence of a greater exploitation of montane species (mostly represented by ibex, accounting for 40% of the NISP) (Fig. 4).

On a wider regional level, continental records at Lake Enol show a slight temperature increase of 2–4 °C at around 14-13kyr BP^91^ (Fig. 3). There are few comparative isotopic results from this period, with only 3 specimens from El Mirón (in the montane interior of Cantabria, c. 160 km to the east) dating to c.13kyr BP, slightly later than the specimens from La Riera. The δ^13^C values are very similar to those observed previously in the sequence, although the mean δ^15^N value is slightly lower at this time (Table 5; Fig. 3). At 14kyr BP, the El Pindal Cave δ^13^C and δ^18^O records show lower values, but increase again at 13kyr BP^28,91^, indicative of changing conditions. The NGRIP record shows the end of GI1, where a slight decrease in δ^18^O values, indicative of a cooler environment^24^ (Fig. 3). There are no “Northern Guest” benthic foraminifera between 15-12.5kyr BP (Fig. 3), suggesting warmer sea bottom temperatures, and this phase is part of a longer trend of declining *N. pachyderma sin* percentages^31^.

#### Interpretation

The shift in the δ^13^C values of the red deer between Levels 23 and 24 appears to be a product of colder, and possibly drier environmental conditions. Positive and negative correlations are seen between temperature and δ^13^C values^81^, and elevated δ^13^C can be indicative of drier conditions^59,60^ (also suggested by the small increase in δ^15^N values, which typically can become elevated when exposed to more arid conditions). Given that this shift coincides with a cooler period, as marked by the NGRIP δ^18^O at the end of GI1, and wider environmental evidence from the site suggests colder environments, a decreasing in temperature, and possible drier environments are potentially responsible for the increase in δ^13^C values. The increased exploitation of ibex in this level (42% of NISP in Level 24, compared to 13% of NISP in Level 23, Fig. 4), a characteristic also seen in the Solutrean levels, indicate a tendency to hunt greater quantities of montane animals during these more rigorous conditions. This zone reflects a short duration of time, with few contemporary isotopic specimens from other sites available to date (Table 5, Fig. 3). It is possible that La Riera was occupied in favour of other sites due to the prevalence of ibex that had found a niche in order to succeed in the area, even during periods of environmental stress.

### Zone 4: Azilian (Levels 27 and 28: 12-9kyr BP)

With the transition to the Azilian (Level 27) a dramatic decrease in the δ^13^C values is evident for both main game species, with mean values becoming more negative at this point, by 1‰ for red deer and 1.5‰ for ibex, and stay at these lower values throughout the Azilian and Asturian (Levels 27–29) (Table 2, Fig. 2).The δ^15^N values of both species remain at a consistent level in comparison to the previous levels sampled (Table 2, Fig. 2).

Between Levels 27 and 28 (c. 12-9kyr BP) a shift in δ^15^N was observed in red deer. The mean δ^15^N value in Level 28 is 0.8‰ lower than Level 27 (Table 2, Fig. 2), and the populations show a statistically significantly difference (*p* = 0.02, Table 3). Values remain at this lower level within Level 29. A similar effect is also observed within the ibex, with the mean δ^15^N value being 0.9‰ lower, although this is not statistically significant(*p* = 0.15), potentially due to the smaller sample size available for Level 28.

The palynological record shows a small increase in arboreal woodland species such as oak, elm and hazel, in addition to ferns (*Polypodium vulgare*), suggesting a warmer, more humid environment during Levels 27 and 28^34^ (Fig. 3). Dormouse (*Glis glis*), which inhabits more temperate and moist environments, was present^37^. Macromammal assemblage continue to show dominance in the exploitation of ungulates that inhabit mixed and wooded environments, notably both red deer and the appearance of wild, for the first time, in the sequence within Levels 27 and 28 boar (Fig. 4). An increase in animals exploited from open landscapes is evident (notably bovines accounting for 10% of the NISP in Level 28)^37^ (Figs. 3 and 4).

Other isotopic records from the Cantabrian Region are more limited for this temporal period. The mean value of specimens from Antoliñako Koba (c. 190 km east on a mountaintop near the coast) is 0.7‰ lower in Z4 than the mean values seen in Z1, the only other zone for which data are available from this site (Table 5). At El Mirón within Z4, the mean δ^13^C values and δ^15^N values are indistinguishable from to those seen within Z3 and no statistically significant differences were observed between populations between these zones, and it is only within Z5 when decreased δ^13^C values and increased δ^15^N values are evident at El Mirón (Table 5), indicative of changing Holocene conditions. Between 12-9kyr BP. Lake Enol temperature estimates average temperature of 10 °C, which is warmer than observed earlier in the sequence^27^. At El Pindal Cave from 12kyr BP onwards, there is a continual increase in the δ^13^C record.

The NGRIP data for this period is marked by the brief lowering of δ^18^O values marking the Younger Dryas, followed by the large increase in δ^18^O values that indicate the onset of the Holocene (Fig. 3). No “Northern guests” are seen in the Basque continental shelf cores, indicating warmer sea bottom temperatures^31^. The slow, but steady decline in the *N. pachyderma sin ‰* and the steady increase in the δ^18^O_lob_ record is also indicative of warmer sea temperatures.

#### Interpretation

This zone corresponds with the start of the Holocene and is marked by more negative δ^13^C values of both species at La Riera. This pattern appears to extend further and is seen in the herbivore bone collagen record within wider Europe datasets, where after 14kyr BP there is a trend towards decreased δ^13^C values^49^. This change may in part be related to increased concentration in atmospheric CO_2_ alongside a possible increased water availability^108,109^, as further evidenced by the increased presence of arboreal pollen, and forest dwelling species within the wider environmental records at the site. The lower δ^15^N values within Leve 28 support this interpretation, as there is a correlation between lower δ^15^N values and wetter environments^69^, which are also reflected in El Mirón later, in Z5. This indicates a possible delayed response in nitrogen to the changing climate^52^. The increased suite of vegetation present in the pollen record, indicates greater habitat diversity, which in turn is reflected in the change in the suite of animals exploited in Level 28. In Level 28, a shift towards exploiting larger areas of the landscape was observed, with the inclusion of plain species (bison) which are infrequently present in assemblages before this point (Fig. 4). Diet in general becomes broader, with larger quantities of marine shells appear in the malacological record^86^. The Holocene in Asturias, and in the whole Cantabrian region, is marked by a change in the suite of macrofauna fauna present, with reindeer, bison and cave bear disappearing and increasing numbers of forest- dwelling species such as wild boar and roe deer^38,110^, which are more suited to these temperate environments.

### Zone 5: Asturian (Level 29: 8.6-6.5 kyr BP)

Within Level 29 (a shell midden deposit), the δ^15^N ranges for both species are larger than seen in any other period (red deer 2.5‰, ibex 2.7‰, Table 2). This indicates a greater inter-individual variability within both red deer and ibex populations. The δ^13^C values remain at a similar level to those observed within Z4.

The pollen record for Level 29 is similar to that of Level 28, with the presence of abundant tree pollen, dominated by hazel, with oak, elm, alder and birch also represented. In addition, higher instances of ferns and slightly higher proportions of arboreal pollen are observed, indicative of a moist and temperate environment^34^ (Fig. 3). Sediment composition also suggests that the level was formed under temperate and humid conditions^85^.

Red deer of El Mirón (9kyr BP) have higher δ^15^N with mean value of 4‰, which is 1.2‰ than seen within Z3, and δ^13^C are 1‰ lower (Table 5). Lake Enol has records of water temperature of 12–13 °C at 8.5 to 8kyr BP, before dropping slightly to 10–11 °C (Moreno *et al*. 2014). In the El Pindal Cave speleothem record, there is a decrease in δ^13^C and δ^18^O during the last part of the record at c. 8kyr BP indicative of a cooler environment than seen previously in the sequence and may be reflecting the 8.2kyr BP event^27^.

The higher δ^18^O values in the NGRIP record are part of the continued trend for warmer Holocene climates^24^ (Fig. 3). The marine core influx of benthic “Northern Guest” species c. 9.3kyr BP and 7.8kyr BP indicate cooler sea bottom waters and the sharp decrease in δ^18^O_lob_ is thought to relate to the early Holocene cooling events^26,31^.

#### Interpretation

This level corresponds to the shell midden (‘conchero’) that capped the infilling of the site. The larger ranges in the δ^15^N values might indicate that animals were consuming plants from a broader environmental area, encompassing a range of isotopically different locations. These larger ranges could be related to the accumulation of midden deposits over a long timescale, resulting in a wider variety in values observed. The longer spans of time represented in the midden layers, in comparison to the lower levels, could have resulted in a higher variation in values, representing a series of environmental fluctuations within levels. An alternative explanation for the diversity in stable isotope values is that the animals hunted from this level came from isotopically diverse areas of the landscape and could be indicative of procurement of resources from larger catchment areas. The concept of base-camps being used, with hunting parties likely travelling within a 15–40 km walking distance of the site has been proposed as a model of site use^111^, a with coastal-inland movements aimed at maximising exploitation of local resources^41^. Animals gradually accumulated, from different parts of the landscape at La Riera, could reflect exploitation from several isotopically distinct locations.

## Discussion

Throughout the sequence at La Riera a series of changes in environment were detected in the bone collagen δ^13^C and δ^15^N isotopic records, and other environmental proxies showed shifts in both animal niches and human hunting habits, which has global significance for enhancing our understanding of the importance of European refugia during the Late Pleistocene.

At the beginning of the sequence, during the Solutrean (20.5-17kyr BP, Zone 1), conditions appeared to be cold and arid, with ericaceous plants dominating the landscape. Throughout Z1, during relatively hostile Late Glacial conditions, ibex were able to adapt their niche and survive. The high-relief environments surrounding La Riera enabled them to successfully alter their feeding behaviour in response to those adverse environmental conditions. Niche partitioning of herbivores is a characteristic observed throughout the Pleistocene, both isotopically^45,66,112^ and using dental microwear^113^. Archaeological studies have indicated that niche partitioning may not necessarily be predictable through time^45^. Adapting feeding habits in relation to changing environmental conditions can be a valuable survival strategy, reducing competition for resources, potentially reflecting mosaic environments. Despite the pronounced effects of the climatic severity, the suite of environments surrounding La Riera provided alternative habitats for ibex, outside of the niche of the red deer, enabling them to thrive and thus to be available to be hunted extensively by humans near the site^37,90^. The predictable presence of ibex surrounding La Riera environs may have been one of the major attractions of hunter-gatherer populations during times of hardship.

Studies of Late Glacial macromammals imply that local hunting strategies were adapted to the local conditions^114^, and at La Riera the results indicate that the availability of ibex during this time of climatic turmoil may have allowed hunters to adapt their strategies. This may, in part explain, the success of this region for human and animal populations during the LGM as attested by genetic studies^14,15,17^ in validation of the classic archaeological refugium theory. Both red deer and ibex were crucial to the survival of Solutrean populations of Cantabrian Spain, although with fluctuations as to which species was dominant^21^. The environments surrounding La Riera cave would have offered a reliable source of game, a highly favourable characteristic for Late Glacial populations^21^ and may have acted as a hub or base camp for Solutrean bands inhabiting the area.

The climatic improvement that occurred at the end of the LGM throughout the Lower-Upper Magdalenian (16-15kyr BP, Zone 2) is represented by a shift in the niche of the ibex, causing them to have more similar δ^13^C and δ^15^N values to those of the red deer. This change in values also coincides with a change in vegetation away from ericaceous plants, towards more steppic conditions, and suggests that plants in the landscape could successfully support both species. The consistency in both the δ^13^C and δ^15^N isotopic values throughout this period suggests that, the Cantabrian region offered environmental predictability which may explain why this area was so densely occupied with a remarkable increase in the number of archaeological sites, estimated at 10–15 per millennium^10,11,13,19,20^. Stability in the resource availability, during this newly improved climate and environment, would have been a major draw of the region to human populations.

Colder conditions observed in Level 24 (13.5kyr BP, Zone 3) coincide with a shift towards greater consumption of ibex, a trait observed during the Solutrean at La Riera when the environment was also characterised as being cold. This suggests that La Riera inhabitants were able to adapt their hunting strategies towards targeting different prey when conditions were harsher, indicating human resilience. This cool spell was not reflected in three other sites in the Cantabrian region, where stable isotope records were available and may reflect series of microenvironments or resource ‘patches’ facilitating survival of different species in around the region effectively, buffering the varying climatic conditions. This environmental buffering, enabled by differential expressions of climate on a local level across the region, may have allowed animals and humans to shift locations depending on resource availability. The movement of populations and the expansion of wider territories into both, coastal and inland areas, has long been discussed as a model of occupation in the region^115–117^. By having the flexibility to change location, even during times of environmental hardship, the occupants of the Cantabrian Region refugium were able to survive, and even thrive. La Riera was a location that offered abundant game, even during colder more challenging conditions and demonstrates its importance in the network of sites in the local and regional framework.

The final parts of the sequence at La Riera show two key trends. During the Azilian, in Zone 4 (12-9kyr BP), warmer and wetter Holocene conditions are recorded. These conditions continue during the Asturian (Mesolithic) (8.6-6.5kyr BP, Zone 5) with another trend, that is, an increase in the ranges of δ^15^N values in both animal species. Hunting animals from a wider catchment area, causing a larger spread in the data, and may indicate increased hunting ranges. Expanded hunting ranges could be due to a need to feed larger numbers of people, to maximise food returns. An increase in human demography is seen at this time, supporting this hypothesis. Other changes in diet are also seen, with a wider dietary breadth in the region throughout the Late Upper Magdalenian, Azilian and Asturian, with a greater number of marine species being exploited, along with lower ranked ungulate prey (such as roe deer), species that are more dangerous and difficult to hunt (e.g. wild boar) and with high percentages of juvenile animals^40,118^. Additionally, more intensive processing of carcasses, such as marrow cracking of small bones, such as phalanges is seen in La Riera from the Upper Magdalenian onwards^37^, and at the site of Las Caldas in central Asturias at this time^119,120^. In the wider region, stone boiling to maximise nutritional output from carcasses^121^ is observed. There have been questions as to whether this dietary change is result of environmental change or population pressures and over-hunting^90,118,122,123^. The evidence from earlier in the La Riera sequence suggests that the major ungulate species (particularly ibex) being exploited by humans at the site were quite resilient and able to adapt to changing conditions, even during times of harsh climatic conditions. This suggests that human population pressures on ungulate populations in Upper Magdalenian-Azilian times may have been responsible for this dietary change. The increase in number of sites per millennia recorded in the archaeological record, throughout the Late Pleistocene^19,20,22^, attests to the opportunities offered by the region, even in the face of increased population size. Whilst the environment played a role in the suite of species on offer during the Pleniglacial, the move towards hunting lower ranked, less sustainable prey is likely a product of an increased human population, causing pressure on resource availability.

During the Mesolithic (Asturian) in the Cantabrian region, populations start to abandon much of the interior, moving away from the densely forested inland areas with limited ungulate prey, likely due to over- exploitation, and towards the coastal ecotone strip with newly formed estuaries and inlets^124,125^, something which is seen at La Riera, where coastal resources (i.e., fish, molluscs, crustaceans) became major and easily collected food resources^86,90,126^. This phenomenon has also been observed across the wider Cantabrian region, as attested by the zooarcheological record and by technological specialisation^118,126–130^, and a marked increase in the number of coastal sites emerging in the Mesolithic^131^. A decrease in the exploitation of larger mammals is evident in the region^118^. It is thought that demographic pressures, more than (or at least in combination with) environmental conditions themselves, were likely ultimately responsible for the shift in human diet^41,90,132–134^. Intensive carcass processing, and increased exploitation of smaller species and aquatic resources has been identified across Europe during the latter stages of the Upper Palaeolithic and is often attested to population growth^135,136^, suggesting that the observations at La Riera are consistent with trends occurring happening on a pan-European scale at this time.

## Conclusions

The large-scale climatic changes that dominated the Late Pleistocene have been detected within the bone collagen record δ^13^C and δ^15^N correlating with the wider proxies within the archaeological sequence at La Riera, thus providing insights into the conditions experienced by humans occupying this refuge region. The isotopic study has shown that the climatic conditions were responsible for some of the affecting changes in vegetation, and thus plant and faunal δ^13^C and δ^15^N values. Behavioural changes associated with those fluctuating conditions were also integral in producing many of the trends observed in the isotopic record at La Riera, influencing both animal feeding behaviour and human hunting strategies. Humans occupying the site and animals living in the locality of La Riera showed the capacity to adapt to these new environments, and to change their feeding behaviour, demonstrating their resilience. Animals might have altered their ecological niches by feeding in different environmental zones, that were readily available for human exploitation, buffering the effects of harsher climatic conditions. The predictability of resources in the environs of the site, even during more environmentally unstable times, may explain why this region was routinely occupied throughout the Upper Palaeolithic. La Riera was just one site in a dense network of Upper Palaeolithic sites in the Cantabrian Region, and by moving between sites and exploiting locally available sources during different seasons and environmental conditions, humans succeeded to survive, and even thrive during the harsh Late Glacial conditions. During the end of the Upper Palaeolithic, in the Azilian and in the Asturian, environments become wetter and increased hunting ranges are seen, in addition to more intensive carcass processing and dietary diversification. Human and animal populations were able to cope with adapting to more hostile conditions earlier in the sequence, suggesting that these changes during early Holocene may be linked instead to pressure on resources, due to an increased human population size. The multi-proxy approach used here shows the value of integrating lines of evidence to characterise how global climatic changes were expressed on continental and local levels and how these affected the Late Glacial human populations, and has wider applications for understanding the importance of European refugia during these times of climatic turbulence.

## Materials and Methods

### Bone sampling strategy

Specimens were sampled from the archive collections held at the Museo Arqueológico de Asturias in Oviedo. The archaeological material was organised relating to the original numerical level attributions assigned during excavation which are inverse to the levels discussed and explained in the site monograph^82^. In this study, archaeology excavation levels have been correlated to the levels as discussed throughout the original publication^82^ to facilitate comparison with the wider palaeoenvironmental datasets available from the site. Levels with secure stratigraphy, pertaining to discrete temporal units, spanning the Solutrean to the Asturian period, were selected to provide insights into changes in environment through time.

Red deer (*Cervus elaphus*) and Spanish ibex (*Capra pyrenaica*), the most consistently consumed species throughout the sequence at the site^37^, and within the wider Cantabrian region during Late Pleistocene^118^ were selected for analysis. Mature individuals, as identified from state of bone fusion were selected for analysis to prevent any animals with elevated δ^15^N nursing signatures^137,138^, which can mask environmental signatures. Bones with evidence of anthropogenic alteration were targeted, including specimens exhibiting cut marks and green fractures indicative of marrow extraction, to ensure that the palaeoenvironmental evidence was directly linked to periods of human activity.

### Analytical methods

Collagen extraction was undertaken following a modified Longin^139^ method with an ultra-filtration step^140^. Samples ranging between 0.7–0.9 g were drilled and cleaned using abrasion. Samples were demineralised in 0.5 M HCL at 6–8 °C for between 3–10 days, and then washed three times using de-ionised water before being gelatinised in a weak acidic solution (pH3 HCL) at 70 °C for 48 hours. Samples were filtered using 5–8 μm Ezee® mesh filters (Elkay Laboratory Products), before being ultra-filtered to separate out the larger > 30ka collagen chains. The > 30ka fraction was then frozen and lyophilized for 48 hours. Between 0.2–0.45 mg of collagen was weighed into tin capsules for analysis of stable carbon (δ^13^C) and nitrogen (δ^15^N) isotope ratios. Specimens were analysed in duplicate, using a Delta XP mass spectrometer coupled to a Flash EA 2112 elemental analyser. The δ^13^C values and δ^15^N values are reported relative to the V-PDB standard and AIR standards. A series of international and internal standards were used to calculate analytical error which was ± 0.1‰ (1σ) or better. The mean difference observed between duplicate measurements was 0.03 for δ^13^C, and 0.01 for δ^15^N. Data were not normally distributed, meaning that the nonparametric Mann-Whitney U test, with a post-hoc Holm-Bonferroni correction^141^ was used. A *p*-value of < 0.05 or less was deemed to be indicative of a statistically significant result.

### Comparative environmental and climatic proxies

Macromammalian faunal information from La Riera^38^ was compiled and plotted as %NISP of the major prey species exploited to create Fig. 4, excluding minor species such as carnivores represented by occasional fragments. The full faunal spectrum from La Riera is provided in Supplementary SI2. For the creation of Fig. 3 species were sorted by habitat type for the creation of Fig. 3. Red deer and wild boar were included in the mixed/forest category. Montane species were, predominantly, represented by ibex, which preferentially occupy rocky cliffs and slopes such as in the nearby Sierra de Cuera. Species attributed to open landscapes included horse and bovids (aurochs and bison which are often not possible to speciate further). Microfaunal remains at the site were scarce, likely linked to sampling strategies used to recover materials (i.e., hand-picking from fine-mesh water-screen residues, rather than from flotation or dedicated stratigraphic column sampling), meaning that the assemblage may not be entirely representative of the full suite of species inhabiting the landscape. Environmental indicators species were identified including *Glis glis* (dormouse), which is characteristic of more temperate environments and *Microtus oeconomus* (root or tundra vole), indicative of cold (and humid) environments, and only their presence is indicated in Fig. 3. The microfaunal data from La Riera are also provided in Supplementary SI3. Pollen data available from la Riera^34^ were collated into categories that have been combined into Mediterranean forest, Atlantic forest, heath and steppic environments (see Supplementary SI4). A summary of evidence and interpretations of the sedimentary record is included in SI5 ^85^.

Regional continental environmental indicators were used to explore how the local environmental conditions observed at La Riera relate to wider landscape trends. Lake temperature estimation, based on Ca precipitation in Lake Enol (Picos de Europa, eastern Asturias), provides an additional indicator of temperature changes throughout the study period^27^ (see Supplementary SI7). Speleothem δ^18^O and δ^13^C records from El Pindal cave (also located in Asturias) were collated^28^ (see Supplementary SI8). Additional bone collagen δ^13^C and δ^15^N studies from across the region have been included for comparative purposes. Data was available from La Paloma, roughly 100 km west of La Riera^49^, El Mirón Cave^52^ (on the northern edge of the Cantabrian Cordillera in eastern Cantabria, about 160 km east of La Riera and 20 km from the present shoreline at 260 m a.s.l.). Two more sites from the Basque region were also included: Antoliñako Koba^26^ and the palaeontological site of Kiputz IX^48^ (all shown in Fig. 1). Data from red deer was included to enable cross-comparisons, but ibex data were not available (see Supplementary SI6). Isotopic data were plotted according to the radiocarbon ages rather than archaeological levels, to enable direct comparisons with results generated from La Riera.

Records available from the continental shelf, including the percentages of *Neogloboquadrina pachyderma sin*. Ostracods, δ^18^O values from *Globigerina bulloides* and interpretations were available^31^ using data from cores KS05-10, KS05-05, and KS04-16 (see Supplementary SI9). This enables a regional, offshore perspective of environment for comparison, and are the closet available records to this study region. These provide an indication of regional temperature changes.

Finally, NGRIP data δ^18^O values available^25^ were plotted at 500-year intervals in Fig. 3 to show general climatic trends throughout the study period (see Supplementary SI10) facilitating correlations of site based, and local data. Global climatic events^142^ are also marked within Fig. 3.

## Supplementary information

Supplementary Dataset 1-10.

## Data Availability

All data generated or analysed during this study are included in this published article, and within the associated Supplementary Information Files SI1–10).
